# ‘People that suffer or have been through it know the answers’: stakeholders’ perspectives on improving healthcare systems for end-of-life care in chronic obstructive pulmonary disease

**DOI:** 10.1186/s12913-023-10431-9

**Published:** 2023-12-20

**Authors:** Amanda Landers, Suzanne G. Pitama, Suetonia C. Palmer, Lutz Beckert

**Affiliations:** Department of Medicine, University of Otago, Christchurch 2 Riccarton Ave, Christchurch Central, Christchurch, 8011 New Zealand

**Keywords:** Chronic Obstructive Pulmonary Disease, Healthcare network, Quality health care, Healthcare systems

## Abstract

**Background:**

Chronic obstructive pulmonary disease (COPD) is a progressive and disabling lung condition with a high mortality. Our research has shown that health care for end-of-life COPD is poorly integrated. The aim of this study was to involve people with end-of-life COPD, their support people and health professionals in the design of healthcare services to help improve the delivery of care for advanced COPD, including informing system-level quality improvement.

**Design:**

We conducted a focus group study involving stakeholders of healthcare services: people with end-of life COPD, support people, bereaved support people, and community- and hospital-based health care professionals.

**Methods:**

We conducted qualitative analysis using deductive structural coding, and then inductive descriptive and pattern coding. Analyses were triangulated by investigators. The research positioned people with end-of-life COPD, their support people and health professionals as experts in healthcare services. Critical theory and Actor-Network theory informed the analysis.

**Results:**

Seven focus groups involving 74 participants reported their experiences of end-of-life care for COPD. Five themes related to healthcare systems responses to improving care quality were identified: governance, system integration, resource design and development, standardisation of processes, and communication.

**Conclusion:**

Stakeholders provided multiple healthcare system-level responses to end-of-life care in COPD that could inform healthcare service design and clinical quality improvement.

## Background

Chronic obstructive pulmonary disease (COPD) is a long-term lung condition that affects millions of people around the world [1]. People with COPD commonly experience progressive physical and social limitations due to breathlessness, with an unpredictable trajectory and intermittent acute deterioration [2]. The expanding number of health professionals involved in the care of someone with end-of-life COPD, as their health needs become more complex, may lead to fragmentation of service delivery [3]. End-of-life care may be defined as the period of time closer to death, likely the last year, where the focus is more on comfort.

The involvement of people with end-of-life COPD and their support people in quality improvement of health services is gaining traction across the world [4]. Health systems all over the world identify the need for new and disruptive ways of delivering quality healthcare to people with complex health conditions to improve patient outcomes [5]. To include health consumers in healthcare design, end-users have been invited to participate in stakeholder workshops, project committees, and mapping exercises [6]. However, the systematic evaluation of people’s lived experiences of the healthcare system, including support people and health professionals, to inform healthcare redesign is uncommon [7]. A gap exists between the ideology of “patient-centred care” and the drive for a health system design that integrates patient and support people expertise within service delivery changes [8].

The aim of this study was to conceptualise the experiences of stakeholders involved in the health care of people with end-of-life COPD including patients and their support people, to understand stakeholders’ perspectives on improving healthcare systems.

## Methods

We conducted a qualitative focus group study to explore the values, perspectives and experiences of stakeholders in health care services for end-of-life COPD, with a focus on the system-level factors that affect the quality of care. The research team consisted of a respiratory physician, Indigenous psychologist researcher, nephrologist and palliative care physician, all with extensive experience in qualitative methodology. A diverse research advisory group including a Māori general practitioner, health funding manager, Pasifika nurse manager and respiratory service leader, provided oversight to the research team at design and evaluation stages. The full description of the methodology of this study has been published elsewhere [9].

We conducted this study in Aotearoa New Zealand, which has a population of just over five million people. The population includes 17.4% identifying as Indigenous (Māori) and 6.9% identifying as Pasifika peoples [10, 11]. Health care in Aotearoa New Zealand includes the provision of free health services in secondary care and subsidised primary health care according to income, coexisting illness and age. In 2022, a new health system governance was commenced with centralisation of 20 independent health boards responsible for healthcare within a geographical location into a single entity (Te Whatu Ora Health New Zealand) with the goal to improve efficiencies across regions and achieve consistent clinical access and outcomes for the population.

In this context, we used focus groups to evaluate end-of-life care services for people with COPD. Focus groups were chosen as they facilitate both the similarities of individual experience, and their variations. People with end-of-life COPD and their support people, and health professionals, were positioned as experts in healthcare service delivery. Support people included spouses, partners, siblings and friends, as invited by patient participants. Interactive discussion within focus groups was facilitated via targeted questions about participants’ values, perspectives, and experiences of end-of-life care. The interview schedule was developed from a systematic literature review and discussed with Māori researchers. The Canterbury region of Aotearoa New Zealand was the selected site, as the research team had strong links with the respiratory department, and networks in the community. Canterbury has approximately 600,000 residents that represent 12.8% of the whole New Zealand population, with 9.4% identifying as Māori and 3.2% identifying as Pasifika Peoples [12].

Focus groups lasting 45 to 90 min were conducted in either a community or a hospital setting, depending on the preferences of the participants. Focus groups comprised:

1. Two Non-Māori/Non-Pacific community focus groups for ***peoples with end-of-life COPD and their support people.***
2. One Māori community focus group for ***Māori peoples with end-of-life COPD, their support people and a health professional.***
3. One Pasifika community focus group for ***Pasifika peoples with end-of-life COPD, their support people and health professionals.***
4. One group of ***bereaved support people*** requested an opportunity to form a specific focus group.
5. Two focus groups involved ***community and hospital-based health professionals (health professionals)***, including allied health professionals, aged residential care carers, primary care practitioners, and respiratory specialists.

Māori and Pasifika health researchers each led their community focus groups, respectively and co-facilitated the discussions to assist in providing culturally safety and discussion in preferred languages. AL, a palliative care physician, led the other focus groups with relevant co-facilitators.

At the commencement of each focus group, the research team displayed a diagram of the existing healthcare network for end-of-life COPD healthcare reported in published qualitative studies [13]. The facilitators then asked participants to discuss whether this diagram reflected their experience in the health system. Specific enquiry questions explored participant experiences of healthcare integration. The focus group transcripts were audio-recorded and professionally transcribed verbatim. The use of Te Reo (Māori language) and Pasifika languages were encouraged during the interview and translated by the research team and facilitators during the transcription process.

We used critical theory and Actor-Network theory as the methodological framework for the overall data analysis. Critical theory enabled a critique of health systems by exploring power relationships and their impact on power differentials, and the identification of actions that might address imbalances in power [14]. Critical theory was utilised to describe how participants were reporting the equilibrium of power among stakeholders including government, healthcare funders, health professionals, community actors, and patients and their support people. Alongside critical theory, the Actor-Network Theory was used to explore complex social systems comprised of human interactions and inanimate objects such as resources, information technology and devices [15]. The Actor-Network Theory was used to explore the depth and impact of the socio-political landscape on end-of-life care services for people with COPD. The research team utilised this methodological framework to validate participate voices and experiences as experts of health systems, including availability and distribution of resources, and to present solutions to support health advancement for these communities.

The transcriptions were uploaded into NVivo software (Version 12, QRS International, Melbourne, Australia). The first coding cycle used the deductive analysis method of structural coding (undertaken by AL) where data was organised into categories using each focus group question [16]. This paper reports the analysis of data identified as related to health systems [17]. Descriptive coding was then used to organise the data into codes inductively. The second cycle of coding identified relationships between codes into categories through pattern coding [16]. A further cycle of pattern coding organised the categories into themes. All cycles of coding and the criteria for codes, categories and themes were peer reviewed and discussed amongst all authors until consensus was achieved.

## Results

**Fig. 1:**
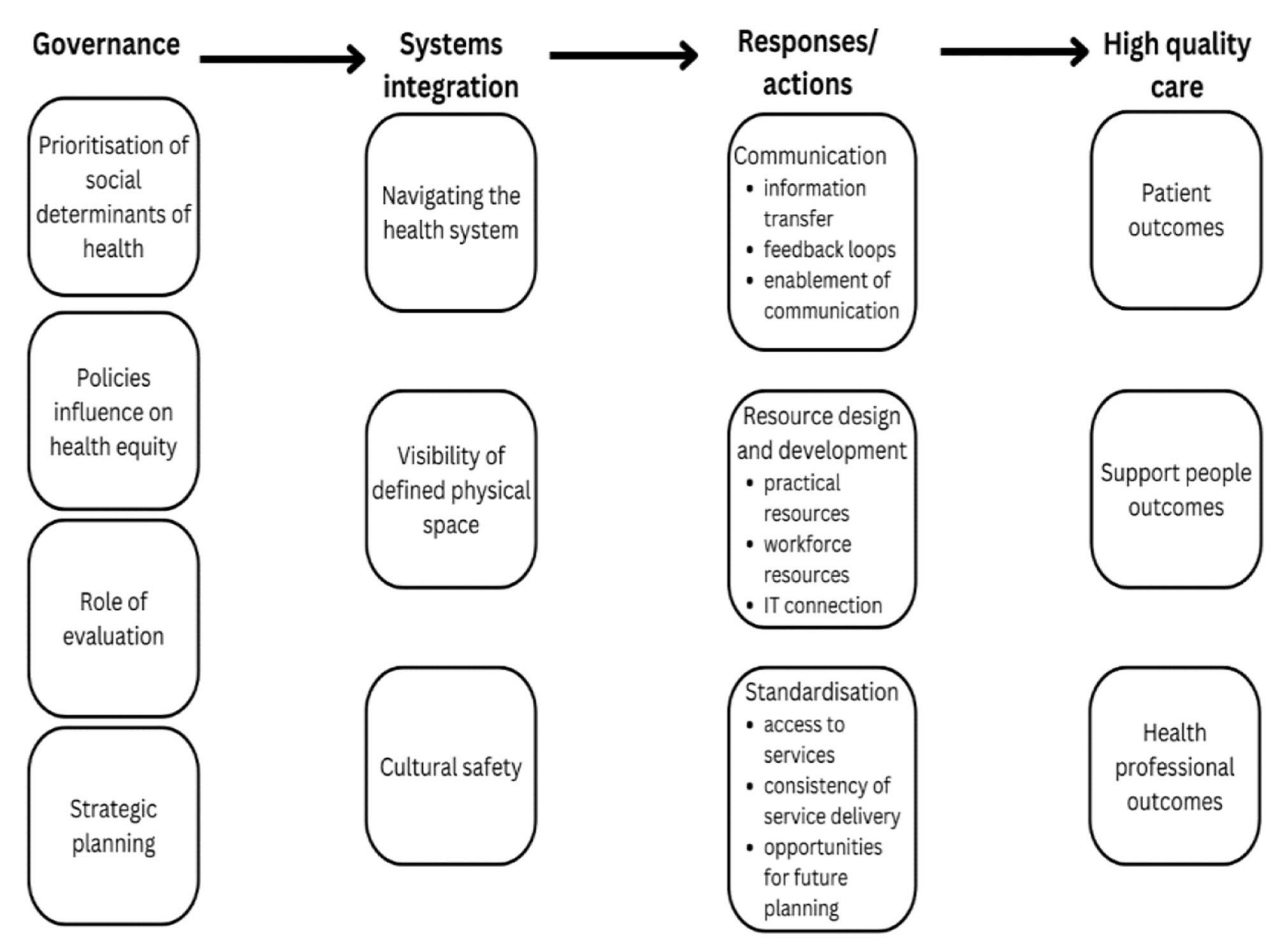
Concept diagram of recommendations from patients with end-of-life COPD, their support people and health professionals in the improvement of end-of-life care. This diagram shows how stakeholders highlighted the important components of governance which linked to system integration which lead to responses/actions and finally high quality care for patients, support people and health professionals

Seven focus groups involving 74 participants were included. (Table 1) Five themes were identified: system integration, communication, resource design and development, standardisation of processes, and governance. A conceptual diagram was generated to reveal links between themes. (Fig. 1)

**Table 1:** Characteristics of participants in the seven focus groups (n = 74)

Focus Group	Participants	Patients	Support People	Health professionals
1	Pasifika peoples with severe COPD and their support people	1	3	5
2	Bereaved support people		5	
3	Māori peoples with severe COPD and their support people	5	1	1
4	Non-Māori, non-Pasifika peoples with severe COPD and their support people	8	3	0
5	Hospital-based health professionals			16 Nursing n = 4 Medical n = 8 Allied Health n = 4
6	Non-Māori, non-Pasifika peoples with severe COPD and their support people	10	2	0
7	Community-based health professionals			14 Nursing n = 7 Medical n = 4 Allied Health n = 3

### Systems integration

Systems integration was defined by participants as an opportunity to improve patient care through service cohesion and a co-ordinated approach to care. Participants described navigating the system, visibility of services, and cultural safety as the key indicators of health system integration.

#### Navigating the health system

Participants with end-of-life COPD and their support people reported difficulties navigating the healthcare system including knowing the correct questions that enable access to appropriate services at the right time. Participants with end-of-life COPD and their support people discussed how the role of a health navigator could guide them through the complexities of primary, secondary and community-based services. Participants proposed that the health navigator could be a person with working knowledge of COPD, the health and social systems. Pasifika participants preferred health navigators from within their own communities, with whom they have relationships and who understand their cultures.

*I think we do need an advocate within our healthcare system and actually our community. All of that middleman that actually shares the info from hospitals to your GP and back, that sort of thing* (FG 1, Pasifika support person).

Health professional participants acknowledged their difficulties navigating community-based services and coordinating these to support patients. Health professional participants described their roles in advocacy and navigation as part of their work. Health professional participants were forthcoming about their current limitations of time and understanding of the illness, and also reported the need to invest in community workers to ensure timely health navigation for patients with end-of-life COPD.

#### Visibility of defined physical space

Participants with end-of-life COPD and their support people desired a physical entity dedicated to COPD services to bring clinicians, patients and support people together in one place. Participants described their aspirations for a hub staffed with COPD and chronic respiratory illness experts. Participants with end-of-life COPD discussed how this hub could enhance their care, by providing required services and where appropriate referrals to other services would be coordinated. Participants described how health navigators would be part of the core service at the hub to help with accessing social services and other non-health government departments.

*A hub that one stop shop where all the knowledge is held. All the links to all the services can be explained to the patients as they come in. It’s like when you get diabetes and you go to the diabetes clinic and you’re put in touch with every service. Dieticians, you know that kind of thing. It should be like that. Someone should hold all of that knowledge. Some body, a body.* (FG 2, Bereaved support person)

Health professional participants recognised the disconnection of the numerous services, teams, and organisations involved in the current health care system, and especially noted this disconnection between primary, community and secondary care services. Health professional participants reported health advancement for patients with end-of-life COPD required those working within the health care system to have a better understanding of each other’s roles, responsibilities and visibility to manage and support those impacted by this condition.

#### Cultural safety

Māori participants and their support people expressed the desire to be at the forefront of any system change affecting their communities. These participants reported the aspiration to involve whānau (family/support network) in decision-making about their own care, and to influence the attainment of beneficial health outcomes.

*why doesn’t someone just turn around and say, “Okay, why don’t we come together and say what we need, let’s see what systems we need to go in to get that,” because I believe whānau should be at the forefront of this. Whānau should be sitting there. Any of these decisions and every part of that should have a whānau that suffers from COPD because they know the answers. And I believe that. I believe that that’s why I do what I do because whānau sit at the front with us. People that suffer or have been through it know the answers because they’re the ones that will know* (FG 3, Māori patient).

Both Māori and Pasifika participants and their support people described the need for health delivery systems to better align with their worldviews and values, to increase engagement and acceptability of services that could effectively support health equity. Health professional participants who identified as Pasifika were concerned by the lack of diverse Pasifika nation representation in the health system, and hence cultural competency was more tailored towards some communities over others. Non-Māori, non-Pasifika health professional participants also described the need for culturally competent resources for Aotearoa New Zealand to support a more knowledgeable workforce and community.

**Table:** 

Proposed changes from participants for system integration	
1.	Health navigator with knowledge of COPD, health and social systems
2.	Physical hub dedicated to bringing medical and support systems together
3.	Culturally-safe support systems, cultural competent workforce and resources

### Communication

Communication was explained by participants with end-of-life COPD, their support people and health professional participants as the information transfer of clinically relevant information, which led to efficient service delivery. Health professional participants described the importance of feedback loops between identified COPD healthcare stakeholders as critical to the provision of high-quality end-of-life care in COPD. Access to communication devices and translators were also signposted as critical to delivering services for those with end-of-life COPD.

#### Information transfer

Participants with end-of-life COPD and their support people described encounters in the acute care sector of the healthcare system where relevant clinical information did not appear to be communicated between medical teams. These participants reported that disruptions in information transfer led to multiple retellings of the same story and delays in treatment. Consequences of these disruptions included missed medications, and the inability of health professionals involved in the care to view proposed treatment plans available to the person with end-of-life COPD.

*But even the system there itself is I think, quite flawed. I know it’s really frustrating sometimes when you continually get asked the same question, but I understand why when you go from department to department in the hospital, all different viewpoints. Somebody can pick up something different to help you. But they just don’t communicate. (FG4, patient)*

Episodes of lost information between health services in different settings were also reported by participants with end-of-life COPD and their support people participants. These participants supported the need for relevant clinical information to be visible in real-time to all health professionals involved in their care. Support people described encounters where time-sensitive patient information had not been communicated between members of the healthcare team, which led to inappropriate and unnecessary visits in the community, duplication of referrals, and undue stress.

*a week after [...] passed away, there was a knock on the door and [...] walks in, ‘hi, where is he?’*

*Hadn’t been told.*

*Yeah, and she is his palliative care nurse but they were through [health care provider A] and they hadn’t been told. Of course, I’d told [health care provider B] and I forgot to tell them and so…*.

*Communication.*

*It’s really bad.*

*100% it is really bad. Terrible.* (FG 2, Bereaved support person)

Health professional participants reported that when clinically relevant information was available between teams their knowledge of the patient’s current status improved. These participants reported that their decision-making benefited by having information from multiple sources.

#### Feedback loops

Health professional participants expressed the desire to have a robust feedback mechanism after sending a referral to another service or organisation. They described how the feedback could be an electronic acknowledgement of the referral, stating when the patients were likely to be seen, and, where appropriate, a preliminary management plan. Health professional participants stated that feedback from each referral would reassure them a plan was in place for the patients with end-of-life COPD. These participants considered that accountability checks in the system to ensure information had been sent and received could improve information flow.

*People get you know discharged when they’re well enough to leave hospital and then you may make these referrals and you never really know did that the referral get through. It’s on the patients and the GP -did it happen? (FG5, DHB HP)*

Health professional participants perceived that an ability to receive feedback on the progress of patients across primary, secondary and community services after referral would reassure them that patients with end-of-life COPD and their support people were continuing to receive appropriate care. Participants with end-of-life COPD and support people stated they wanted communication systems that were timely, accurate and relevant to their communities.

*With this Pasifika team I’d like an in-patient-based one and then a community-based one that communicate with what’s happening in both ideas, so we get this full information from primary and secondary, so on and so forth.* (FG 1, Pasifika support person)

#### Enabling communication

Health professional participants which were based in community health settings, provided examples of how patients wanted to communicate with their healthcare team when devices such as cell phones were not available, or patients had little access to email or the internet. An example included the ability to talk with a person when ringing GP practices, instead of dealing with automated message services, which saved time and reduced stress for patients.

Pasifika participants, their support people and health professional participants discussed the complexity and lack of access to ensure appropriate translation services for each Pasifika language group. Health professional participants who identified as Pasifika described services in other parts of the country that were working well and suggested these could be reproduced locally.

*They have really good translation services up there because when I did a course there, you probably heard it as well, they said just tell them what language you want your thing translated in, send it to them and they’ve got translators on hand and they will type it all up. We don’t have that here. (FG1, Pasifika HP)*

**Table:** 

Proposed changes from participants for communication	
1.	Relevant clinical information visible in real time to all health professionals
2.	Robust feedback mechanisms after referral to another service
3.	System in place for patients to contact health professionals in person
4.	Appropriate translation services for non-English speaking people

### Resource design and development

Resource allocation was outlined by all participants as related to practical considerations such as transport and equipment, workforce and information technology systems, all of which could improve quality of life. Some participants suggested ways of developing services and adjusting policies that would streamline their care.

#### Practical resources

Some participants reported a dearth of practical resources which created barriers to: self-management of COPD, accessing services logistically and the ability to retain physical function. Some participants also suggested increased funding and support for home-based rehabilitation services that would teach patients and support people about COPD, build relationships, and enable more effective follow-up.

Some participants also described difficulty in accessing equipment such as home aids secondary to criteria based on age, setting and illness.

*Yeah well, he got the stool and that when he was in the hospital. But now he’s been discharged he gets nothing.* (FG5, Support person)

Participants with end-of-life COPD and support people expressed the importance of being able to access the right equipment, at the right time for more effective self-management at home. They needed to know what was funded, and what they would need to source themselves. Further resources to provide this support was advocated for within the focus groups.

#### Workforce resource

Participants with end-of-life COPD and support people described current disruptions to the healthcare system which included services with waiting lists such as counselling, respiratory physiotherapy and respite care. Health professional participants discussed the need for an increased community-based allied health workforce including respiratory physiotherapists and psychologists to support patients with end-of-life COPD. All participants desired increased investment into the home-care workforce as organisations were struggling to provide care in certain areas of the region due to staff shortages.

*It’s just the healthcare system. Another thing, I feel like when I first started getting them it was only casual for six weeks or something as you do and all that, and they’d ring up and say, “We’re going to get somebody,” and all this, and nobody would come. Then I might get them once and then I try to get through to them, I say, “Look, nobody’s comes,” “Oh no, you only had it for six weeks and we couldn’t find anybody and now your six weeks is up.”* (FG6, Patient).

Health professional participants discussed that the acute shortage of primary care physicians in the community led to an increased workload in primary care. Health professional participants, who were community based, discussed how they had practical experience with life coaches attached to their practice, and suggested this extended the capability and capacity of a GP practice out into the community. These health professional participants also reported that the utilisation of services such as a home-based pharmacist medicine reconciliation and explanation supported people with end-of-life COPD in their own environments. Participants with end-of-life COPD and support people reported that services willing to visit at home were useful, improved adherence to medications and treatment plans, and built connections with families.

#### Information technology connection

All participants described significant disruption to high-quality care was due to a lack of integrated information systems across the healthcare system. Some progress was reported by health professional participants, who were hospital-based, in IT system connection. These participants suggested further gains in connectivity could be made by allowing all health care professionals, including those in the community and social system, to access IT platforms for relevant patients.

*And the IT has to be you know if the government were going to invest in anything it would be the one physical thing that they can invest in. Because that’s what connects those people. And getting NGOs and things access to that information.* (FG 5, Hospital-based HP)

Participants with end-of-life COPD and support people validated in their discussions the importance of health professionals having the ability to access information across all settings in the health system. Health professional participants expressed the desire for a National Health IT system with appropriate investment to better support patients with end-of-life COPD in their care and treatment.

**Table:** 

Proposed changes from participants for resource design and development	
1.	Increased funding and support for home-based services
2.	Ability for health professionals to access correct equipment for patients
3.	System for health professionals to access community-based allied health for patients
4.	Investment into a National Health IT system

### Standardisation

Standardisation was defined as uniform procedures or practices in health that guided organisations and health professionals. Standardisation was signposted by all participants to include delivery of services that provide consistent care, and enable patients and support people to proactively plan for the future.

#### Delivery of services

People with end-of-life COPD and support people participants reported the unpredictable delivery of services such as specialist respiratory assessment. These participants described delivery of services as a lottery system, which they reported was dependent on variables such as talking with a certain person or having specific circumstances that opened up other opportunities for care. Health professional participants discussed current processes in place that were helpful to assist them understand how services were delivered such as Health Pathways, a co-designed set of localised pathways for illnesses and presentations. Participants with end-of-life COPD and their support people also suggested that an ideal way forward would include having standardised triggers for people with end-of-life COPD who presented with specific risk factors that led to pre-determined community supports and referrals.

*at a certain age if you identified as a smoker on your classification list it should be automatic, not rather we think about and we think, oh, you’ve got a bit of a cough go and see [...]. There should be something like a …There’s outcomes that you can do that can make a difference, and if that means then that the lifestyle stuff kicks in as well…* (FG 7, Community-based HP).

Health professional participants suggested a standardised assessment tool as a mechanism of ensuring all options that aligned with best practice and that were locally available, were offered to people with end-of-life COPD and their support people. Health professional participants discussed the need for adjusted processes that enabled the importance of clear referral criteria for different services so that time was not wasted, or referrals declined without explanation.

#### Consistency of service delivery

Participants with COPD and their support people reported that service delivery after a diagnosis of end-of-life severity appeared erratic and lacked a clear systematic plan. Health professional participants recounted experiences of patients with advanced COPD having multiple admissions, with varying levels of support and no consistent follow up. These participants discussed ways to achieve consistency across the acute care setting which included a person with end-of-life COPD being admitted to the same team if appropriate, and/or a flag system in which relevant team members would be automatically notified of an admission. Health professional participants considered this would allow better efficiency and continuity of care.

*There could be I think we have a system in place in palliative care where all patients that are flagged as having seen us in the past, we’re alerted that they’re back in hospital. So that we can poke our nose in and just see whether they need us earlier rather than waiting for two days to be given a referral that would have benefited seeing them sooner.* (FG5, Health professional)

Support people participants also suggested standardised tools such as a flowcharts for staff that would ensure the same options and services occurred for all people with end-of-life COPD, and would better provide a framework to ensure patients and their support people are given correct and timely advice.

#### Opportunities for future planning

Participants who were support people shared perspectives that future planning for end-of-life care happened too late, which led to the patient being too unwell to have meaningful discussions about their individual needs. Support people participants also discussed important decisions such as place of care and the burden these difficult decisions placed on them. Health professional participants discussed the importance of both an acute care plan and an advance care plan, to help people with end-of-life COPD and support people navigate the best options for health care. Bereaved support participants and health professional participants discussed that the experience of care would be strengthened by people with end-of-life COPD being offered acute care plans and advance care plans earlier in the process

*there’s no acute care plan or advanced ACP they’re not done by practice nurses. Like with.*

*Well, they are at some places.*

*They are being done by practice nurses.*

*But not consistently in end stage when those discussions need to be had.* (FG5, Health professional)

People with end-of-life COPD and their support people, and bereaved support participants described the empowerment felt by undertaking the planning process. The complex role of confidentiality and information sharing was also raised in relation to the development of advance care plans.

*The advance care plan, we should all have one. […] you take it with you wherever you go, you have written down but you’ve got to get it when the person’s alive. We did it with X and I’ve got it with me. […] It was written in a better way, more a legal sort of a thing but you don’t have to have a lawyer. […] wherever we went, […] it would make our job easier* (FG2, Bereaved support person).

**Table:** 

Proposed changes from participants for standardisation	
1.	Development of standardisation triggers to community referrals
2.	Clear referral criteria for referral to different services
3.	Flowcharts for staff to ensure the same options offered to all
4.	Standardisation of offering acute and advance care plan discussions

### Governance

Governance captures the entities and people that have the power to change or control the health system. Governance was seen by all participants as integral to improving the quality of care for people with end-of-life COPD including prioritisation of the social determinants of health and the development of equitable policies for access to services. People with end-of-life COPD, support people and health professional participants identified that a clear strategy was crucial, which could be effectively monitored to ensure ongoing quality assurance processes e.g., evaluation.

#### Prioritisation of the social determinants of health

People with end-of-life COPD, their support people and health professional participants discussed how the current health system aligned to a biomedical approach, which was disease focussed. All participants expressed a desire for a health system with key design features to include an increase in health promotion, health education and the social determinants of health. In relation to the determinants of health, some participants reported the impact of specific housing needs on people with end-of-life COPD, with the need for better housing standards to reduce exacerbations of COPD and maintain longer periods of wellness.

*Housing is another thing. Someone that has COPD, they cannot just live in any state or any housing. It cannot be below 17 degrees. You’ve got to have double glazing and it’s not being picky, but it’s the things, cos without those things, their hospital and their exacerbation is more and more…*.

*I’ve learned so much with my brother’s disease and trying to help other people, because those small things like a heat pump and double glazing and a warm place is so important to someone with COPD.* (FG 2, Bereaved support person)

Pasifika support people and health professional participants further expressed the administrative challenges in securing appropriate contracts to provide tailored culturally appropriate services that could address the psycho-social-cultural determinants of health. Participants reported that culturally appropriate services effectively reduced patient hospital admissions.

#### Policies influence on health equity

All participants described policies that created barriers and inequities for patients and their support people. Participants with end-of-life COPD and support people described the challenges of obtaining access to equipment such as shower stools, hospital beds and wheelchairs for patients under 65 years of age. Health professional participants also reported discrepancy in their ability to access services before a patient with end-of-life COPD was diagnosed as palliative and end-of-life. All participants expressed frustration with the current system and desired to see a more equitable service in place.

*we had probably eight months from when he was in hospital until when the GP put him into palliative care and then we got the shower stool and the wheelchair, and I could take him for a walk in the wheelchair. We couldn’t get access to anything before then, because he was under 65 and there was too much paperwork. That was the response.*

*And that’s the same with me and […]* (FG 4, Support person).

Health professional participants reported pathways in the system which created inequality, including financial burdens such as ambulance costs which were funded in some areas of the health system and not others. Participants with end-of-life COPD and support people reported struggling to cope financially, and found it difficult to navigate the social welfare system due to a lack of understanding of their illness. Some participants suggested the need to fund models of practice to address communities most impacted by inequities such as; rural, Māori, Pasifika, younger cohorts, and support people with full-time work commitments. Pasifika support people and health professional participants discussed the value of including churches and different community groups in policy design as they provide the best way to reach communities with health messages and promotion.

#### Role of evaluation

Health professional participants discussed the importance of the whole-of-system approach to health, equitably distributed across all patient groups, and the role of evaluation in this process. They described the effort of health service designers in the creation of new teams and services for patients, mainly with long-term care needs such as end-of-life COPD. Health professional participants also discussed the different models of care they had witnessed in the healthcare system such as palliative care services becoming episodic instead of constant in the care of a person with life-limiting illness. However, they acknowledged the lack of evaluation to inform the ongoing evidence and performance of these services. They also discussed the insufficient evaluation of new services and models of care from a patient and cultural perspective, and wanted this built into any new project.

*I mean we’re trying to I suppose there has been a real push in Canterbury around the whole of system approach. So, trying to get things that are not just all targeted on one patient group. but are more distributed but maybe in the keenness to get these things up and running, to actually ask patients and research patients about how they’re working and whether those things are useful for them, or appropriate for them. Never mind culturally appropriate you know all of that element. And we just say we don’t think that that’s actually happened.* (FG5, Health professional)

Health professional participants commented on the role and importance of research to investigate models of care, new services and equity in the provision of end-of-life care services.

#### Strategic planning

Support people and health professional participants mentioned the desire for further strategic planning from the government and health system designers in relation to end-of-life COPD care. Health professional participants reported the need for a National Respiratory Strategy, including cultural and illness prevention elements, to provide a foundation of a future vision for end-of-life care services for people with COPD. Health professional participants also expressed the desire for a one-system approach to health that encompasses all settings and bio-psycho-social elements, with projects that actively promote integration. An example discussed was the creation of an integrated physiotherapy service that crosses boundaries with the regional health sector. Māori participants reported the lack of a cultural strategy plan on actively addressing inequities for Māori with end-of-life COPD. Māori participants discussed their expectation to be at the forefront of any strategic planning or system design with their whānau. Pasifika participants articulated the need for an illness prevention strategy that focused on spiritual, mental, financial and emotional well-being alongside the physical. Pasifika participants believed any strategic planning should include churches, community groups and their families.

*I think there’s a lot of work that needs to be done within the health system and within the supports given and that we need to, hopefully the government understands the worth of families, of churches, of our different community groups and to create that illness prevention strategy that focuses on working within our world view. That’s not just the physical, but also the spiritual, mental, financial, emotional, all those other wellbeing parts of our model.* (FG 1, Pasifika health professional)

**Table:** 

Proposed changes from participants for governance	
1.	Prioritisation of health promotion, education and social determinants of health
2.	Contracts tailored to provide culturally-appropriate services
3.	Ability for health professionals to access services equitably for patients
4.	Increased funding to address communities most impacted by inequity
5.	Adequate evaluation of service provision
6.	Strategic planning for end-of-life COPD services

## Discussion

This study focused on perceptions of end-of-life care for people with COPD. Analysis of people with end-of-life COPD, their support people and health professionals’ experiences and perceptions of healthcare services identified five themes to describe a high-functioning healthcare system: integration, communication, standardisation, resource allocation and governance.

Participants expressed how healthcare delivery could be strengthened through quality improvement specifically for COPD, rather than a wider whole-of-system approach. This approach is supported by evidence showing that when healthcare systems implement large-scale, whole-of-system reforms to maximise health system efficiencies to a whole population [17], they encounter difficulties with sustainability and resource management [18]. Quality healthcare improvements appear to work better when focused on a single disease or population [19, 20]. There is also evidence that stakeholder involvement in the design of these services leads to better outcomes for patients, their support people and health professionals (Fig. 1.) [21].

The system changes proposed by people with end-of-life COPD and their support people differed from those originating from health professionals and management. There were important differences between what patients and their support people viewed as provision of high-quality care in end-of-life care in COPD and the perceptions of health professionals. Design of a health system that enabled navigation for those with chronic, long-term illness was critical to all stakeholders. For participants with end-of-life COPD and support people, a designated health navigator who held knowledge of the system and delivery of services was preferred. In our analysis, health professionals believed they were fulfilling this role and were not aware of this gap in care delivery. Evidence suggests health navigators are associated with positive outcomes, and work effectively to link consumers with the correct services [22]. People with end-of-life COPD and support people also favoured a visible hub where health and social services for those with this condition are delivered holistic care. There is little evidence in the literature evaluating the effectiveness of community-based and -led health organisations. The benefits of such an entity are unknown and should be systematically studied. A central hub for people and families living with pre-diabetes and diabetes is currently being evaluated in a randomised controlled trial that may inform the development of disease-specific centres for delivery of high-quality care [23].

This analysis highlighted disconnection between governments and the health system in end-of-life care for COPD. Participants reported on government health policies that created inequity and a vacuum of strategic vision for high quality care provision. This finding aligns with our systematic review showing very few connections between stakeholders in the healthcare system, and government in healthcare delivery for advanced COPD [13]. A key finding in that evidence synthesis was the lack of priority placed by governments on addressing socio-political determinants of health in the delivery of high quality health care. A positive example of government policy taken on the research finding is the delivery of safe and warm housing to improve health outcomes for people with lung diseases [24]. Our research makes a similar case for improving service delivery to people with end-of-life COPD and their support people.

### Strengths and limitations

Our research team conducted this study in a large region of Aotearoa New Zealand and triangulated the voices of multiple stakeholders. The analysis utilised critical Theory and Actor-Network Theory to prioritise patients, support people and health professionals as experts in end-of-life care for COPD. The research advisory group, including a health manager and public health physician, was consulted through the project and allowed for further analysis of the data. It is difficult to be certain how transferable these results are to other health systems. Participants from at-risk backgrounds were not included, and cultural aspects were not included in this article.

## Conclusion

Stakeholders provided multiple healthcare system-level responses to end-of-life care in COPD that could inform healthcare service design and clinical quality improvement.

## Data Availability

The datasets generated and analysed during the current study are not publicly available due to participants only consenting for the data to be utilised for this research project. Data was included from Indigenous Peoples who retain sovereignty over the information. Please contact author AL if requesting data from this study.
